# Mast cell activation mediates blood–brain barrier impairment and cognitive dysfunction in septic mice in a histamine-dependent pathway

**DOI:** 10.3389/fimmu.2023.1090288

**Published:** 2023-02-01

**Authors:** Jianhe Yue, Ying Tan, Renzheng Huan, Jin Guo, Sha Yang, Mei Deng, Yunbiao Xiong, Guoqiang Han, Lin Liu, Jian Liu, Yuan Cheng, Yan Zha, Jiqin Zhang

**Affiliations:** ^1^ Department of Neurosurgery, The Second Affiliated Hospital of Chongqing Medical University, Chongqing, China; ^2^ Department of Neurosurgery, Guizhou Provincial People’s Hospital, Guiyang, China; ^3^ Department of Nephrology, Guizhou Provincial People’s Hospital, Guiyang, China; ^4^ Department of Anesthesiology, Guizhou Provincial People’s Hospital, Guiyang, China

**Keywords:** mast cell, sepsis-associated encephalopathy, neuroinflammation, blood-brain barrier, histamine

## Abstract

**Introduction:**

Sepsis-associated encephalopathy (SAE) is a diffuse cerebral dysfunction resulting from a systemic inflammatory response to infection; however, its pathophysiology remains unclear. Sepsis-induced neuroinflammation and blood–brain barrier (BBB) disruption are crucial factors in brain function disturbance in SAE. Mast cells (MCs) activation plays an important role in several neuroinflammation models; however, its role in SAE has not been comprehensively investigated.

**Methods:**

We first established a SAE model by cecal ligation puncture (CLP) surgery and checked the activation of MCs. MCs activation was checked using immumohistochemical staining and Toluidine Blue staining. We administrated cromolyn (10mg/ml), a MC stabilizer, to rescue the septic mice. Brain cytokines levels were measured using biochemical assays. BBB disruption was assessed by measuring levels of key tight-junction (TJ) proteins. Cognitive function of mice was analyzed by Y maze and open field test. Transwell cultures of brain microvascular endothelial cells (BMVECs) co-cultured with MCs were used to assess the interaction of BMVECs and MCs.

**Results:**

Results showed that MCs were overactivated in the hippocampus of CLP-induced SAE mice. Cromolyn intracerebroventricular (i.c.v) injection substantially inhibited the MCs activation and neuroinflammation responses, ameliorated BBB impairment, improved the survival rate and alleviated cognitive dysfunction in septic mice. In vitro experiments, we revealed that MCs activation increased the sensitivity of BMVECs against to lipopolysaccharide (LPS) challenge. Furthermore, we found that the histamine/histamine 1 receptor (H1R) mediated the interaction between MCs and BMVECs, and amplifies the LPS-induced inflammatory responses in BMVECs by modulating the TLR2/4-MAPK signaling pathway.

**Conclusions:**

MCs activation could mediate BBB impairment and cognitive dysfunction in septic mice in a histamine-dependent pathway.

## 1 Introduction

Sepsis is a systemic inflammatory disease defined as a life-threatening organ dysfunction caused by a dysregulated host’s reaction to infection. It affects a shockingly high number of patients, with estimates ranging from 19 to 48.9 million cases per year worldwide, and remains a leading cause of death worldwide (1). Sepsis is also considered the main killer in intensive care units due to higher mortality rates as well as multiple organ infection at the same time (2). The central nervous system (CNS) is one of the first organs impaired by sepsis, and about 9%~71% of patients with severe sepsis could develop sepsis-associated encephalopathy (SAE) (3). SAE is responsible for short-term morbidity, prolonged hospital stay, long-term physical and cognitive impairment, and poses a large economic burden to healthcare systems (4). Exploration of the possible mechanisms of SAE showed that sepsis-associated neuroinflammation is one of the crucial factors in the disturbance of brain function in SAE. During sepsis, endotoxemia and pro-inflammatory cytokines are released systemically, leading to excessive immune cell activation. The mass production of inflammatory cytokines perpetuates a vicious cycle, leading to brain impairment and contributing to the progression of SAE (5). Mast cells (MCs)-associated neuroinflammation has recently attracted attention. The activation of these cells plays important roles in various neuroinflammation models, such as trauma, stroke and neurodegenerative diseases (6–9). Based on recent findings, MCs are among the first responders to injury in the CNS (10). However, the role of MCs in SAE has not been comprehensively investigated.

MCs are crucial immune cells derived from hematopoietic stem cells (11). In the brain, MCs are predominantly located in proximity to the basal side of the blood–brain barrier (BBB), to serve as immune sentinel cells that respond against environmental stimuli (9). It becomes activated with exposure to a diverse array of stimuli, from allergens and antigens to neuropeptides, trauma and drugs (6). The activated brain MCs can rapidly release their prestored and newly formed vasoactive mediators and inflammatory cytokines, such as histamine, tryptase, 5-serotonin, TNF-α and a variety of cytokines (8, 11, 12). Among those inflammatory chemicals, histamine is the most specific. It is a neurotransmitter and an immune modulator, and both roles are involved in aminergic neurotransmission, gastrointestinal functions, inflammatory reactions and immune response (13). In the substantia nigra, histamine acts as a pro-inflammatory mediator. Its injection induces microglial activation primarily by histamine 1 receptor (H1R) activation, which ultimately leads to dopaminergic neuronal death (14). Histamine can also act synergistically with proinflammatory cytokines such as IL-1 and IL-6, to modulate the astrocytic release of neurotrophins, such as nerve growth factor (15). Up to 50% of brain histamine levels in rodents are produced by MCs (12), suggesting that the modulation of inflammation by histamine in the CNS may be linked to MCs activation.

The BBB is a highly selective and semipermeable interface that regulates the molecular flux between the blood and brain and thereby maintains a state of homeostasis in the CNS (16). It mainly depends on brain microvascular endothelial cells (BMVECs) and the tight junctions (TJs) between BMVECs to achieve its barrier function. Its existence and integrity maintenance are vital for the normal function of the CNS (17). BBB dysfunction contributes highly to the pathophysiology of SAE (18). During sepsis, activated BMVECs generate an intravascular inflammatory microenvironment, which includes various adhesion molecules and inflammatory receptors, leading to microglial and astrocyte activation, promoting neuroinflammatory response and TJ disruption and finally resulting in BBB dysfunction (19). As a consequence, BBB deficiencies facilitate the infiltration of neurotoxic mediators and water into the brain, causing cerebral edema and hypoperfusion and promoting and exacerbating neuronal damage in SAE (12). However, the interaction between MCs and BMVCEs is poorly understood. In human umbilical vein endothelial cells (HUVECs), researchers have found that histamine up-regulates the expression of Toll-like receptor 2 (TLR2) and TLR4 and amplifies the endothelial cell (EC) inflammatory responses (20). Thus, we wondered whether sepsis-induced MC activation could promote histamine secretion and induce BMVEC inflammatory responses and BBB disruption.

In this study, we aimed to investigate the important roles of abnormally activated MCs in cecal ligation puncture (CLP)-induced encephalopathy. Furthermore, we hypothesized that sepsis-induced MC activation could promote BMVECs inflammatory responses and BBB disruption, both of which could mediate by MC-secreted histamine.

## 2 Materials and methods

**Figure 1:**
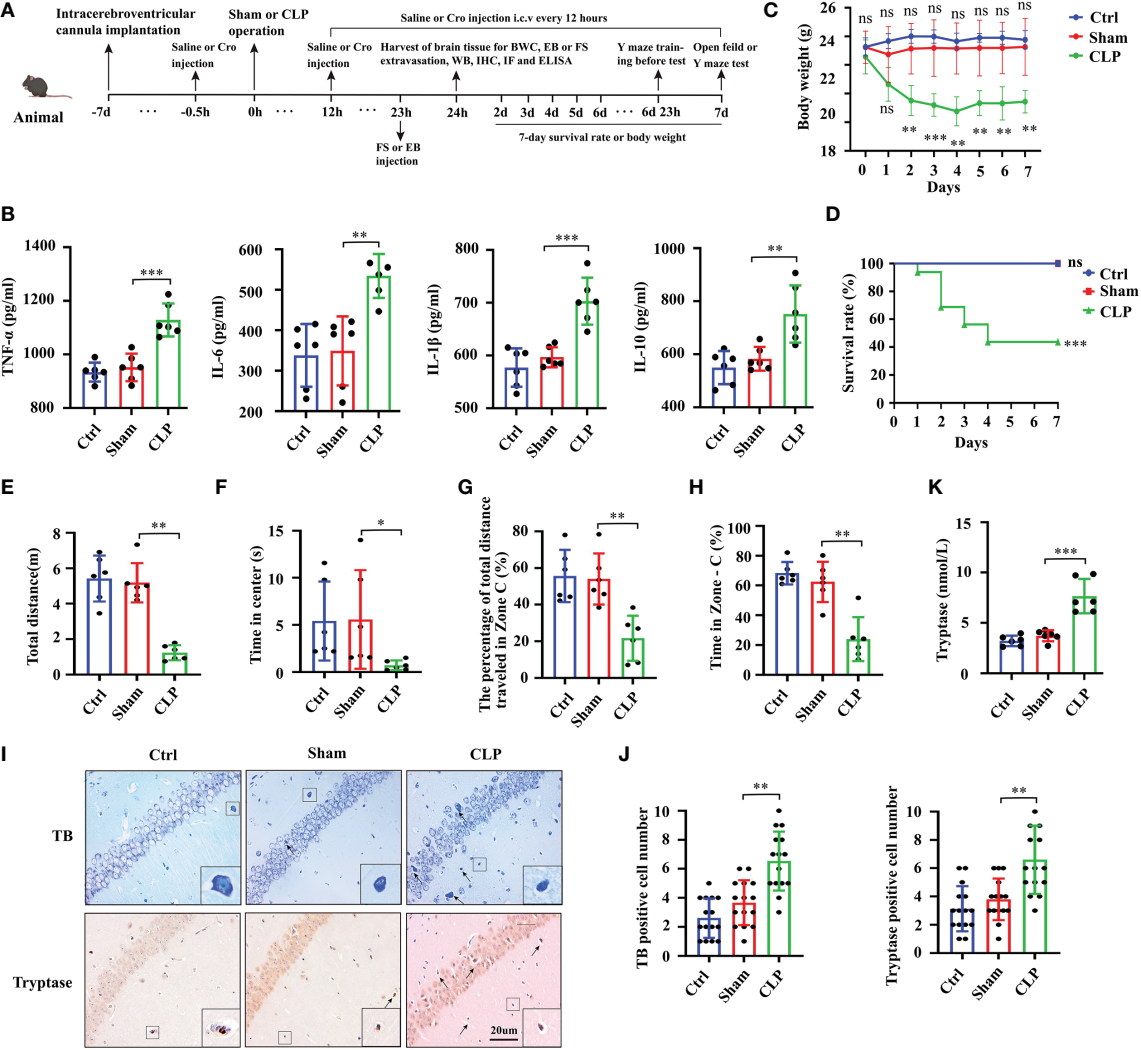
Sepsis-induced neuroinflammation, cognitive deficits and mast cells activation in brain tissues. **(A)** Schematic timeline of the experimental procedures. **(B)** The levels of TNF-α, IL-6, IL-1β and IL-10 in mice hippocampus tissues were detected by ELISA (n=6). Body weight **(C)** and survival rate **(D)** were observed in 7 days following surgery (body weight: n=6; survival rate: n=15). The total movement distance **(E)** and the time in the center zone **(F)** in the open field test, and the movement distance in zone C **(G)**) and the time in zone C **(H)** in the Y maze test were recorded to analyze the cognitive changes (n=6). **(I)** TB staining and tryptase IHC staining were used to detect activated MCs in the hippocampus CA1 region (400x, black arrow indicates MCs, n=6). **(J)** Quantification of TB and tryptase staining positive cells in the hippocampus CA1 region (n=6). **(K)** Levels of tryptase in the hippocampus CA1 region were detected by ELISA (n=6). MCs: Mast cells, CLP: Cecal Ligation and Puncture, SAE: Sepsis-associated encephalopathy, TB: Toluidine blue, IHC: immunohistochemistry. (ns: no significance, * p<0.05, ** p<0.01, *** p<0.001).

All antibodies, reagents, vendors, catalogue numbers, and concentrations or dilution ratios are shown in detail in **Supplementary Table 1**. Schematic timeline of the experimental procedures as shown in **Figure 1A**.

### 2.1 Animals

Male C57BL/6J mice (6–8 weeks of age, 20–25 g) were purchased from the Tengxin Biotechnology Company (Chongqing, China). All mice were fed with standard food and sufficient water in cages under a controllable circumstance (temperature 20°C–23°C, humidity 55%–65%, and a 12h light/dark cycle). The animal program was authorized by the Institutional Animal Care and Use Committee of Guizhou Province People’s Hospital (Guizhou, China).

### 2.2 Cecal ligation and puncture model

SAE was established by CLP as previously described (21, 22). Briefly, the mice were anesthetized with isoflurane, and a 2 cm-wide midline laparotomy was performed to expose the cecum. The cecum was ligated with a 4.0 silk suture at 1/4 distance to the end below the ileocecal flap and then punctured with a 22G sterilized needle. Intestinal contents were pushed out through the perforation site. The cecum was then returned to the peritoneal cavity, and the laparotomy was closed with 4.0 silk sutures. All animals received a dose of antibiotic (primaxin, 0.5 mg/mouse in 200 uL of sterile saline) subcutaneously immediately at the end of surgery. All animals were fed in single cages with free access to food and clean water. In the sham surgery group, the mice were subjected to all surgical procedures, except for ligation or puncture.

### 2.3 Intracerebroventricular cannula implantation and drug administration

Following anesthetization, the mice were placed in the stereotaxic apparatus (RWD life science Co. Ltd., China). In accordance with previous reports (10), guide cannulas (RWD life science Co. Ltd., China) were planted into the right lateral ventricle (coordinates: 0.6 mm posterior to the bregma, 1.5mm lateral, 2mm depth from the dura) and secured to the skull with dental cement. All the mice were allowed to recover in clean cages for 7 days. And handled daily to check the guide cannula and familiarize them with the investigators.

A MC stabilizer, comolyn was dissolved in sterile saline with a final concentration of 10 mg/mL. For the cromolyn treatment group, 2uL of cromolyn was administered intracerebroventricular (i.c.v) at 30 min before the CLP surgery and every 12h after surgery. The other group received 2uL of sterile saline.

### 2.4 Immunohistochemistry and immunofluorescence staining

For IHC analyses, brain tissue sections were incubated for 1 h in 10% bovine serum albumin (BSA) with 0.3% Triton X-100 in 0.01 M PBS and then overnight with anti-tryptase (Abcam, UK) at 4°C. Tissue sections were washed and incubated with anti-rabbit secondary antibody for 1 h at room temperature. Immunostaining was visualized with DAB and counterstained with hematoxylin (Solarbio, China). The slides were scanned using a digital camera (Olympus, Japan). For IF analysis, brain sections or cell slides were incubated overnight with primary antibodies: ZO-1, occludin and Ki67 (Abcam, UK) in blocking solution at 4°C, followed by goat anti-mouse Alexa Fluor Plus 488 secondary antibody (Invitrogen, USA), and then covered with mounting medium containing DAPI (Invitrogen, USA) in a dark environment. Images were captured under a fluorescent microscope (Carl Zeiss, Germany). Images were calibrated for the optical density and then measured the areas and integrated densities. Changes in immunofluorescent intensity were shown (mean fluorescence intensity = integrated densities/areas)

### 2.5 Toluidine blue staining

Brain paraffin sections and cell smears were stained with 1% TB solution (Solarbio, China) following the manufacturer’s protocol. MCs were detected as violet color or different levels of blue color as observed under a microscope. The number of MCs and their degranulation status were determined by their abnormal morphology and the presence of extracellular cytoplasmic granules under a light microscope using 40x or 100x magnification (Olympus, Japan).

### 2.6 Brain water content

The mice were euthanized at 24 h after surgery. The whole brain was instantly collected and weighed as the wet weight, and then dried at 100°C for 24 h to acquire the dry weight. The BWC was calculated using the formula: BWC = [(wet weight−dry weight)/(wet weight)]∗100%.

### 2.7 Fluorescein sodium and Evans blue permeability assay

FS and EB were applied to evaluate BBB permeability as previously described (23). Briefly, 2% FS (Sigma, Germany) and 2% EB (Sigma, Germany) were respectively injected through the femoral vein at 4 mL/kg under anesthesia at 24 h after modelling. After 1 h, all animals were euthanized and transcardially perfused with 0.9% saline for 20 min to remove the intravascular dye. The hippocampus was then dissected from the brain, weighed separately, homogenized in 0.5 ml of 60% trichloroacetic acid and incubated at 4°C for 30 min. After centrifugation (12,000 rpm, 15 min), the FS concentration in the supernatant was measured at OD 440nm and the EB concentration was measured at OD 620nm by a microplate reader (BioTek, USA).

### 2.8 Y maze test

The Y maze consisted of three arms (regions I–III, 30 × 5 × 20cm) that converged to an equilateral triangular central area (region 0) (RWD life science Co. Ltd., China). One arm was chosen as the “start arm”, another as the “novelty arm”, and the third as the “other arm”. Firstly, the animal was placed in the “start arm” with its back to the center, and the “novel arm” was closed. The animal was allowed to explore freely between the two arms for 15 min (training). One hour after the first trial, the animal was placed in the “start arm” with its back to the center, and the “novelty arm” was opened. The animal was allowed to explore freely for 5 min, and its movement trace was recorded. The time spent in each arm of the mouse and the movement distance of the animal in each arm were used as indicators to evaluate spatial recognition and memory. The maze was cleaned with 75% ethanol between tests.

### 2.9 Open field test

The locomotor and exploratory activities of mice were measured in an open-field apparatus. A mouse was gently placed in the center of a plastic chamber (50 × 50 × 40 cm) for 5 min, and its activities were automatically recorded using a video tracking system (RWD life science Co. Ltd., China). The total distance travelled and time spent in the center of the arena were recorded to assess the locomotor activity and spatial exploration of the mouse. The apparatus was cleaned with 75% alcohol between tests to remove any odor cues.

### 2.10 Cell culture and transfection

P815 and bEnd.3 cell lines were purchased from the Chinese Academy of Sciences Shanghai Cell Bank (Shanghai, China). The cells were cultured in Dulbecco’s modified Eagle’s essential medium (Gibco, USA) containing 10% fetal bovine serum (Gibco, USA) and 1% penicillin/streptomycin (Beyotime, China) under 37°C and 5% CO_2_. In brief, over-expression plasmid for H1R was generated by polymerase chain reaction (PCR) and cloning into LentiORF pLEX-MCS vector using a forward primer (5′-ATAAGAATGCGGCCGCGCCACCATGAGCCTCCCCAATTCCTC-3′) and a reverse primer (5′-CCGCTCGAGTTAGGAGCGAATATGCAGAATTCTC-3′). Recombinant lentiviral particles were obtained from the transient transfection of 293T cells following a standard protocol. For transduction, bEnd.3 cells were cultured in six-well plates to 60% confluence and added with viral solutions containing 8 µg/mL polybrene (Solarbio, China). Over-expressing cells were selected under puromycin (Beyotime, China). H1R siRNA was purchased from Obio Technology (Shanghai, China) and transfected into bEnd.3 cells by using RNAi max (Invitrogen, USA) following the manufacturer’s protocol. After transfection for 6 h, the lipid and siRNA complexes were removed, fresh 10% serum medium was added and incubation was performed for 72 h.

### 2.11 Co-culture of bEnd.3 and P815 cells

For co-culture experiments, P815 cells (1 × 10^5^ cells) were planted in the upper chamber of a 12-well Transwell™ plate (Corning, USA) with complete medium and treated with cromolyn for 30 min. The P815 cell chambers were then transferred to a 12-well plate with bEnd.3 cells incubated in the lower chamber, The co-cultured cells were stimulated with LPS (1 μg/mL) for 2 or 24 h.

### 2.12 Cytokine examination

Expression levels of TNF-α, IL-6, IL-1β and IL-10 were quantified using a commercial ELISA kit from Boster Biological Technology (Wuhan, China). Histamine content was tested with an ELISA kit from Elabsecience (Wuhan, China). Tryptase content was tested with a detection ELISA kit from Meimian Biological Technology (Jiangsu, China). All procedures were carried out following the manufacturer’s instructions.

### 2.13 Western blot

Proteins were extracted from homogenized cells or tissues in a lysis buffer (Solarbio, China) containing a protease inhibitor cocktail (Solarbio, China) and boiled in a SDS sample buffer (Beyotime, China) for 15 min. Equal amounts of protein per sample were separated by SDS-PAGE (8% or 10%) and electrotransferred to a polyvinylidene difluoride membrane (Milipore, USA). Membranes were blocked with 5% BSA (Beyotime, China) for 1h at room temperature, and rinsed three times (10 min/time) with 0.5% PBST (0.5mL Tween-20 in 1L 0.01 M PBS). Next, incubated with the specified primary antibodies at 4°C overnight, rinsed three times (10 min/time) with 0.5% PBST, and then 1 h with HRP-conjugated goat anti-rabbit IgG (detailed antibodies were shown in **Supplementary Table 1**), rinsed three times (10 min/time) with 0.5% PBST, followed by detection using an enhanced chemiluminescence kit (Milipore, USA).

### 2.14 Statistical analysis

Statistical analyses were performed with GraphPad Prism 9. All data were presented as mean ± SD and tested for normal distribution by Shapiro–Wilk test. For normally distributed data, differences between the two groups were compared by independent-sample t-tests. Meanwhile, differences among multiple means were assessed by one-way, two-way or repeated-measure of ANOVA, followed by a Bonferroni test whenever appropriate. The survival rate was analyzed by the Kaplan–Meier method and compared by the log-rank test. Statistical significance was set at *p*<0.05.

## 3 Results

### 3.1 Sepsis-induced neuroinflammation, cognitive deficits and MC activation in brain tissues

Singleton et al. suggested that investigators must rigorously control the distance of the cecum ligated to generate consistent mortality and inflammation data when utilizing the CLP model (24). Therefore, we first performed a pre-experiment with 25%, 50% and 75% lengths of the ligated caecum. As shown in **Supplementary Figure 1A**, the mice with 75% of ligated caecum have over 50% mortality rate within 7 days. In body weight, 75% of ligated caecum contribute to a significant drop than of other ligated lengths (**Supplementary Figure 1B**). In addition, expression levels of inflammation cytokines (TNF-α, IL-6, IL-1β and IL-10) in the hippocampus homogenate were also detected using ELISA, and the results showed that 75% of ligated caecum can also cause more inflammatory cytokines production than that of others (**Supplementary Figures 1C–F**). Therefore, 75% of ligated caecum was selected as the most appropriate ligated length to induce SAE for further studies.

To evaluate the neuroinflammatory response during SAE, we detected the expression of IL-6, IL-1β, TNF-α and IL-10 in the serum and hippocampus homogenate following CLP. As shown in **Figure 1B**, all cytokines, either pro-inflammatory cytokines (IL-6, IL-1β, TNF-α) or anti-inflammatory cytokines (IL-10), were substantially increased in the hippocampus tissues at 24 h after surgery (*p*<0.05). The expression levels of IL-6, IL-1β, TNF-α and IL-10 in serum were also significantly increased at 24 h after surgery (*p*<0.05, **Supplementary Figure 2A**). In addition to the severe inflammatory response, the body weight of the CLP mice dropped seriously after surgery (**Figure 1C**). Moreover, several CLP mice did not survive from severe inflammatory response. The 7-day survival rates of the CLP mice decreased significantly compared with those of the sham mice (*p*<0.05, **Figure 1D**). Behavioral manifestations in sepsis survivors were determined using open field trials and the Y maze. In the open field trial, the movement distance for a mouse to explore a new environment significantly decreased for the CLP mice (*p*<0.01, **Figure 1E**). In terms of the time spent in the central region of the open field, the CLP mice spent a significantly shorter time than the sham mice (*p*<0.05, **Figure 1F**). In the Y maze, the CLP mice showed a similarly poor performance because their percentage of total distance traveled in Zone C (**Figure 1G**), time spent in Zone C were remarkably decreased compared with those of the sham surgery mice (*p*<0.001, **Figure 1H**). Additionally, the movement trace of mice has been shown in **Supplementary Figure 2B**. These findings demonstrated that the CLP mice showed notably cognitive deficits.

To check the activation of brain MCs in CLP-induced neuroinflammation, we evaluated MCs in the hippocampus sections at 24 h after surgery. These brain sections were incubated with 0.1% TB and tryptase antibody for MCs detection. The positive cells were stained purple or deep blue (black arrows) after TB staining. The number of positive cells was increased in the CA1 area of the SAE mice hippocampus compared with that of the sham control mice (**Figure 1I**). Quantification data for TB- and tryptase-positive cells are shown in **Figure 1J**. The expression of tryptase in hippocampus tissues was further determined using ELISA at 24 h after CLP surgery. The results showed that the expression level of tryptase increased significantly in the CLP mice (*p*<0.01, **Figure 1K**). These data supported the overactivation of MCs in the hippocampus of mice with SAE.

### 3.2 Cromolyn treatment defended neuroinflammation, improved SAE mice survival, and alleviated cognitive impairment

**Figure 2:**
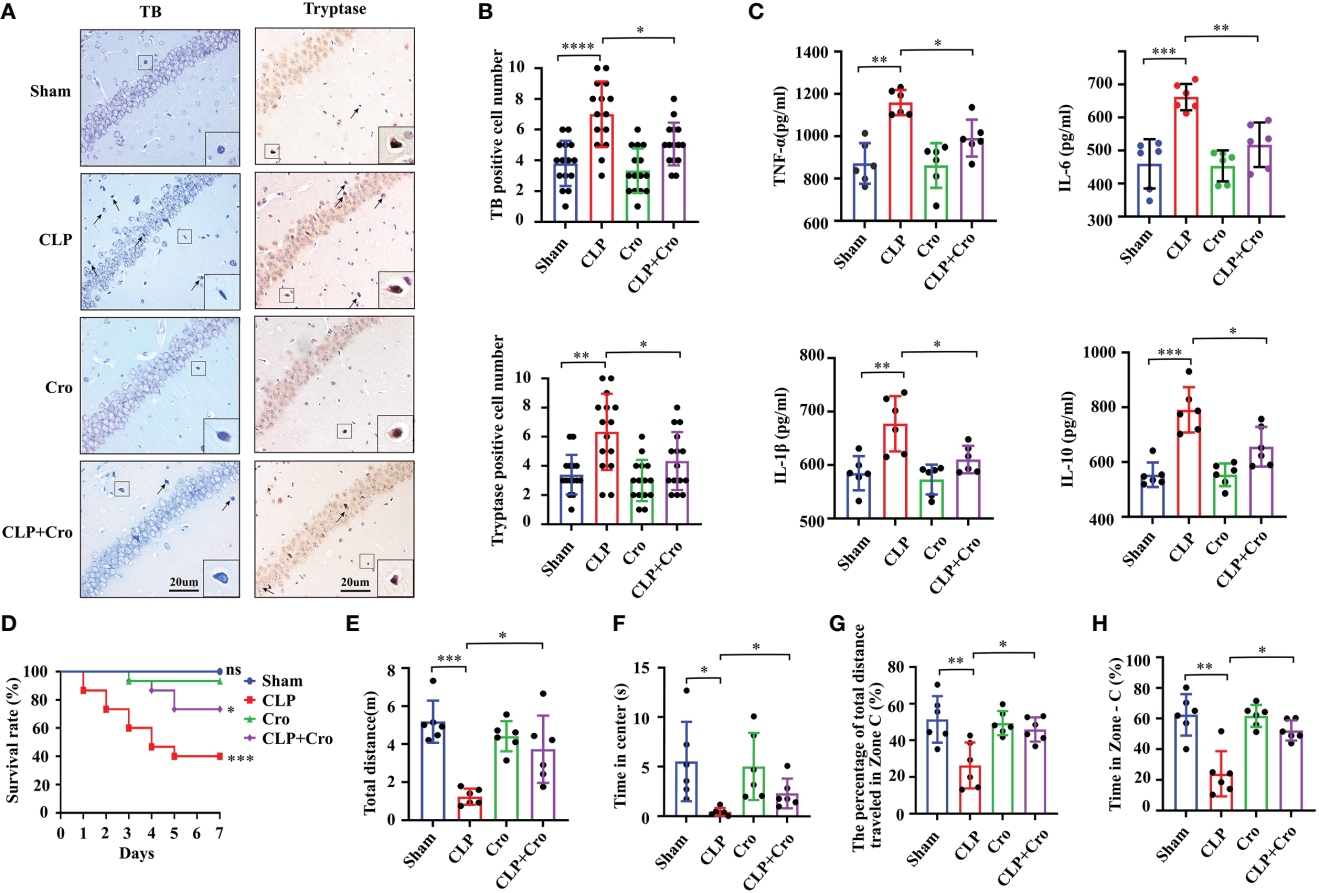
Cromolyn defended neuroinflammation, improved SAE mice survive, and alleviated cognitive impairment. **(A)** Cromolyn inhibited MCs activation in the CA1 region of the hippocampus by TB staining and tryptase IHC staining (n=6). **(B)** Quantification of TB- and tryptase-positive cells (n=6). **(C)** ELISA test showed that cromolyn decreased the levels of TNF-α, IL-6, IL-1β and IL-10 in hippocampus tissue (n=6). **(D)** Cromolyn treatment improved 7-day survival rate in septic mice (n=15). The total movement distance **(E)** and the time in center **(F)** in the open field test, and the movement distance **(G)**, and the time **(H)** in zone C in the Y maze test were increased after cromolyn treatment (n=6). MCs, Mast cell; CLP, Cecal Ligation and Puncture; SAE, Sepsis-associated encephalopathy; TB, Toluidine blue; IHC, immunohistochemistry; (ns, no significance, * *p <*0.05, ** *p <*0.01, *** *p <*0.001, **** *p <*0.0001).

To explore the specific role of MCs in SAE progression, we i.c.v. injected cromolyn, the most characteristic MCs stabilizer, at 30 min before the CLP surgery. The results showed that treatment with cromolyn alone did not affect the activation of MCs in the brain. However, under CLP surgery, cromolyn significantly prohibited the MCs activation in the hippocampus as indicated by TB and tryptase antibody staining (**Figure 2A** with quantification in **Figure 2B**). With the stabilization of MCs, pre-treatment with cromolyn mitigated the expression of intracerebral inflammatory cytokines (IL-6, IL-1β, TNF-α and IL-10) (**Figure 2C**) and improved the 7-day survival of the CLP mice (**Figure 2D**). In addition to the inflammation inhibition, pre-treatment with cromolyn remarkedly increased the movement distance and the time in center in open field trial (**Figures 2E, F**) and improved the performance of the CLP mice in the Y maze (**Figures 2G, H** and **Supplementary Figure 2C**), suggesting that this drug alleviated CLP-induced cognitive impairment. All these results implied that MCs play a crucial role in neuroinflammation and cognitive deficits in the CLP-induced SAE model, and cromolyn can limit the adverse neuroinflammation and cognitive outcomes caused by sepsis.

### 3.3 Cromolyn protected BBB permeability in SAE mice by inhibiting MC activation

**Figure 3:**
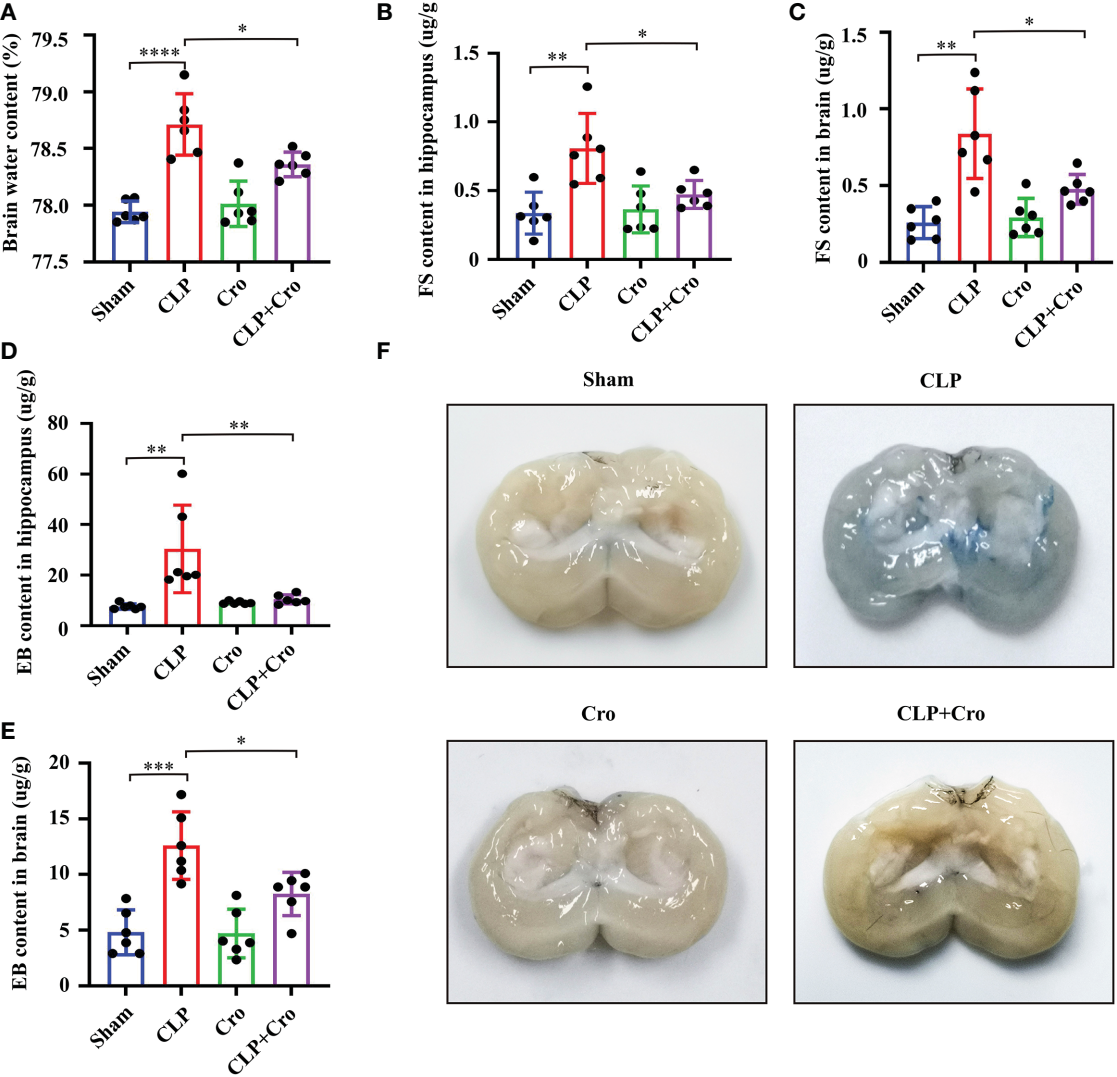
Cromolyn reduced BBB permeability in septic mice by inhibiting MC activation. **(A)** The brain water content was measured. Quantification of the Fluorescein sodium extravasation in the hippocampus **(B)** and the global brain tissue **(C)** Evans blue extravasation in the hippocampus **(D)** and the global brain of mice **(E)** was also detected (n=6). **(F)** The representative images of Evans blue staining of the brains (n=6). MCs, Mast cells; CLP, Cecal Ligation and Puncture; SAE, Sepsis-associated encephalopathy, BBB; Blood–brain barrier, (* p <0.05, ** p <0.01, *** p <0.001, **** p <0.0001).

In this study, we assumed that MC activation mediates SAE progression through BBB disruption. To test our hypothesis, we assessed brain breakage by firstly measuring BWC. At 24 h, BWC was significantly increased in the CLP mice compared with that in the sham mice (*p*<0.0001, **Figure 3A**). Cromolyn significantly reduced BWC in the CLP mice (*p*<0.05, **Figure 3A**). FS and EB extravasation are widely used markers for BBB breakage. As shown in **Figures 3B, C**, the FS levels in the hippocampus and global brain tissue samples were similar in the sham and cromolyn groups. A marked increase in FS levels was observed in the hippocampus and brain tissue in the CLP mice compared with those in the sham mice, and this increase was notably reversed by cromolyn treatment. Additionally, EB permeability tests were performed to assess BBB damage. The results were similar to those from FS extravasation (**Figures 3D, E**), and representative sections for EB staining are shown in **Figure 3F**. These data suggested that MC activation increases BBB permeability in SAE mice, which is partially alleviated by cromolyn treatment.

### 3.4 Cromolyn protected BBB stability by alleviating the disruption of TJs between BMVECs

**Figure 4:**
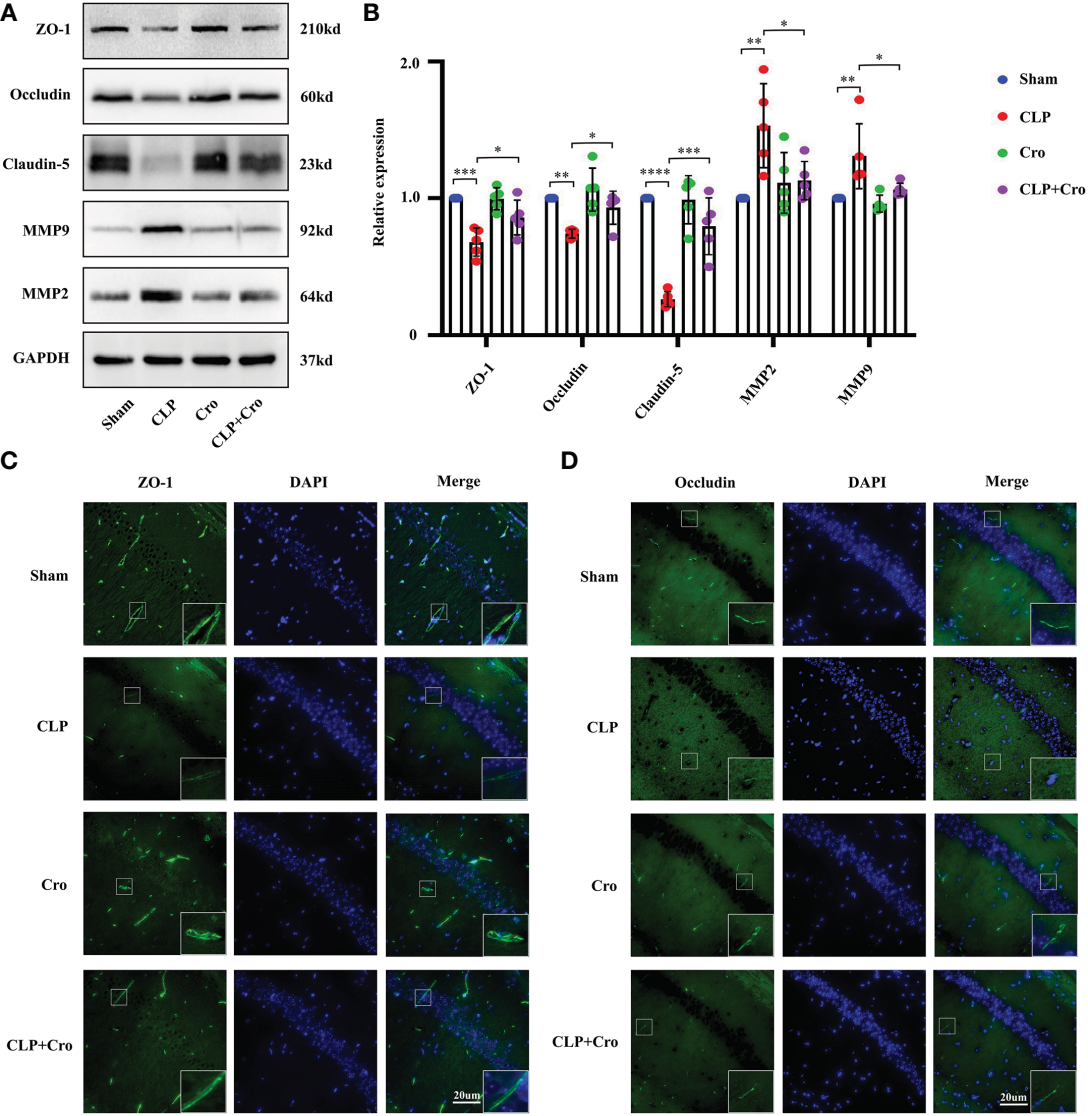
The inhibition of MC activation by cromolyn protected BBB integrity in septic mice by alleviating TJ disruption. **(A)** Expression levels of MMP2/9 and TJ (ZO-1, occludin and claudin-5) proteins in the hippocampus were detected by Western blot (n=5). **(B)** Expression levels of MMP and TJ proteins were quantified and normalized to GAPDH levels (n=5). Expression levels of ZO-1 **(C)** and occludin **(D)** in the CA1 region of the hippocampus were analyzed by IF assay (400x, n=6). MCs, Mast cells; CLP, Cecal Ligation and Puncture; SAE, Sepsis-associated encephalopathy; BBB, Blood–brain barrier; TJs, Tight junctions; IF, immunofluorescence. (* p <0.05, ** p <0.01, *** p <0.001, **** p <0.0001).

The barrier function of BBB is mainly performed by BMVECs and their TJs. ZO-1, occludin and claudin-5 are integral membrane proteins composed of TJ strands and contribute to BBB integrity. Previous studies reported that matrix metalloproteinases (MMPs) can degrade the extracellular matrix (ECM) and destroy the TJ protein (25). Thus, we firstly investigated the expression of MMP and TJ proteins, including MMP2, MMP9, ZO-1, occludin and claudin-5, in the hippocampus using Western blot. As shown in **Figures 4A, B**, the protein expression of MMP2 and MMP9 increased significantly in the CLP group compared with that in the sham group (*p*<0.05). Meanwhile, the protein levels of ZO-1, occludin and claudin-5 were sharply reduced in the CLP group (*p*<0.05). Cromolyn treatment significantly decreased MMP expression and increased TJ protein levels in the CLP group. We then assessed the distribution of TJs between ECs in the hippocampus by immunofluorescence staining. The results showed that SAE-induced neuroinflammation disrupted the distribution of ZO-1 and occludin, spreading them in the form of a dotted appearance and weakening the fluorescence intensity. This finding demonstrates the possibility of losing the protein–protein interaction between TJ proteins might. Meanwhile, the sham group showed uniform staining of TJs (**Figures 4C, D**). Fortunately, Cromolyn spread in the form of a line and enhanced the fluorescence intensity. All these data revealed that MCs are involved in maintaining the integrity of TJs and their activation can lead to TJ cleavage, resulting in BBB disruption. Cromolyn treatment can ameliorate BBB dysfunction by protecting the TJs between BMVECs.

### 3.5 LPS induced the degranulation of MCs *in vitro*

**Figure 5:**
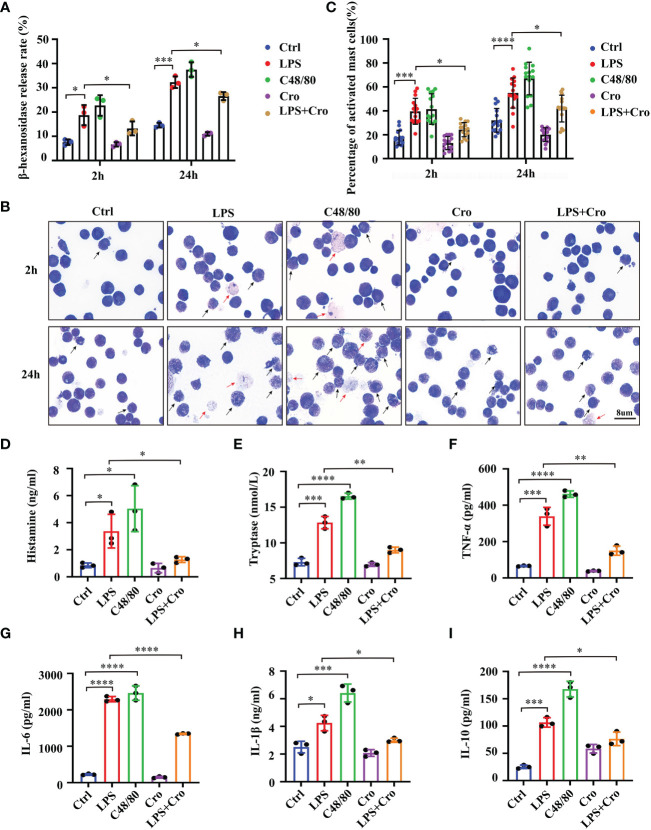
LPS successfully induced the degranulation of MCs, and cromolyn effectively stabilized LPS-induced MC activation *in vitro*. **(A)** Degranulation of P815 cells stimulated by LPS was analyzed by β-hexosaminidase release assay. C48/80 treatment was used as the positive control (n=3). **(B)** Morphological properties of MCs activation were investigated using the TB staining of the cell smears (Black arrow: activated MCs, red arrow ghost-like cell; 1000x; n=3). **(C)** Quantification of activated MCs in TB staining of the cell smears (n=3). ELISA was used to detect the levels of histamine **(D)**, tryptase **(E)**, TNF-α **(F)**, IL-6 **(G)**, IL-1β **(H)** and IL-10 **(I)** in the medium supernatant of P815 cells (n=3). LPS, Lipopolysaccharide; C48/80, Compound 48/80; TB, Toluidine blue. (* *p <*0.05, ** *p <*0.01, *** *p <*0.001, **** *p <*0.0001).

To specifically explore the relationships between MCs activation and sepsis, we performed *in vitro* experiments with P815 cells, a mouse MC line, and quantified the MC degranulation under LPS stimulation. Compound 48/80 (C48/80) is one of the most characteristic MCs activators. C48/80 treatment 007Agroup (10mg/mL) was used as the positive control. As shown in **Figure 5A**, 1mg/mL LPS significantly promoted the β-hexanosidase release rate in P815 cells compared with that in the control group as early as 2 h, the rate was continuously increased at 24 h. Additionally, cromolyn (10mg/mL) treatment significantly alleviated the LPS-induced excessive β-hexanosidase release rate. The morphological properties of degranulated MCs were investigated using the TB staining of the cell smears. As shown in **Figures 5B, C**, the number of degranulated P815 cells was significantly higher in the LPS and C48/80 groups than that in the control group at 2 h. A few “ghostlike cells” appeared in the LPS and C48/80 groups at 2 h (red arrows). Nearly all P815 cells were activated in the LPS and C48/80 groups at 24 h, and many “ghostlike cells” were present. Cromolyn significantly reversed the LPS-induced degranulation of P815 cells. Considering that the release of inflammatory factors is also an important marker of MC activation (26), we additionally evaluated the production of histamine, tryptase, TNF-α, IL-1β, IL-6 and IL-10 in P815 cells after LPS treatment for 24 h. The results showed that the production of histamine, tryptase, TNF-α, IL-1β, IL-6 and IL-10 significantly increased in the LPS group. This trend was partially reversed by cromolyn treatment (**Figures 5D–I**). These data suggested that LPS can successfully induce the degranulation of MCs and that cromolyn treatment can effectively stabilize MCs under LPS stimulation.

### 3.6 Cromolyn treatment protected BMVEC functions from LPS-induced MC activation

**Figure 6:**
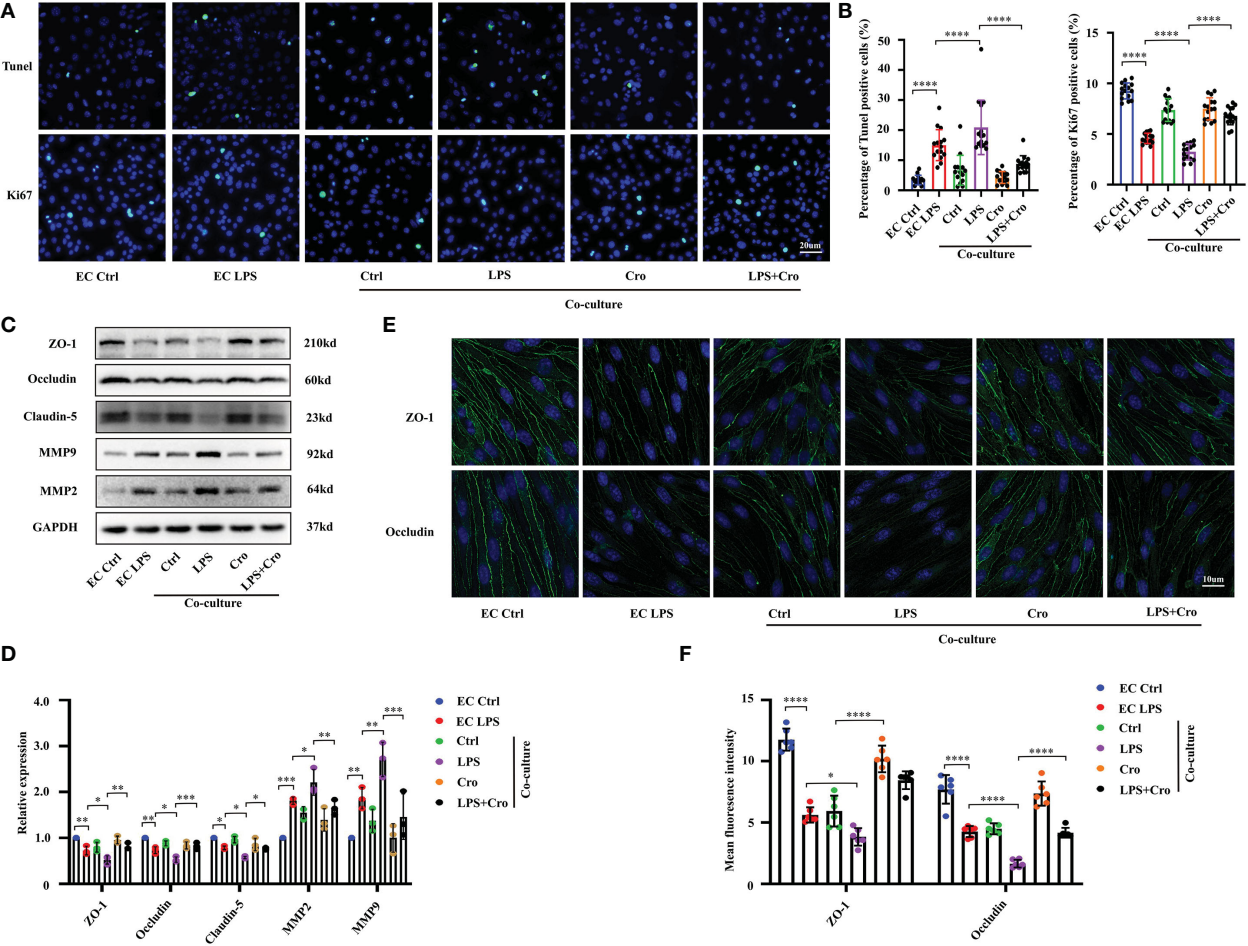
Cromolyn treatment protected BMVEC functions from LPS-induced MCs activation. **(A)** Apoptotic BMVECs and Ki-67-positive cells were examined by Tunel staining and Ki-67 IF staining, respectively (400x, n=3). **(B)** Quantification of Tunel-positive and Ki-67-positive BMVECs (n=3). **(C)** Expression levels of MMP9/2 and TJ proteins (ZO-1, occludin and claudin-5) of bEnd.3 cells were analyzed by Western blot (n=3). **(D)** The expression of MMP and TJ proteins were quantified and normalized to GAPDH levels (n=3). **(E)** Expression levels of ZO-1 and occludin protein in bEnd.3 cells were examined by IF assay (800x, n=3). **(F)**. Expression levels of ZO-1 and occludin were quantified in bEnd.3 cells in IF assay (n=3). LPS, Lipopolysaccharide, MCs, Mast cells; BMVECs, Brain microvascular endothelial cells; TJs, Tight junctions; IF, immunofluorescence. (ns, no significance, *p <0.05, ** *p <*0.01, *** *p <*0.001, **** *p <*0.0001).

Given that the *in vivo* data indicate a relationship between MCs and BBB dysfunction under the SAE model, we attempted to clarify the mechanism of this correlation. Considering that BMVECs and the TJs between them are the most critical factors for BBB integrity, we further explored the effect of activated MCs on BMVECs *in vitro*. The mouse MC cell line P815 and mouse BMVEC cell line bEnd.3 were co-cultured in the Transwell system. Tunel assay and Ki-67 immunostaining were applied to investigate the proliferation and apoptosis of BMVECs under various treatments and examine the effect of MCs activation on the cell viability of BMVECs. The results showed an increase in Tunel-positive cells and a decrease in Ki67-positive cells among the bEnd.3 cells under LPS stimulation (**Figure 6A** with quantification in **Figure 6B**), indicating that LPS could inhibit proliferation and induce apoptosis. In the P815 and bEnd.3 co-culture system, the number of Tunel-positive bEnd.3 cells were further increased, and Ki67-positive bEnd.3 cells were reduced, demonstrating that MCs can promote BMVEC dysfunction under LPS stimulation. To further confirm this conclusion, we pre-treated the co-culture system with the MCs stabilizer, cromolyn, before LPS stimulation and examined the proliferation and apoptosis of bEnd.3 cells. With the treatment of cromolyn, the viability of bEnd.3 cells were partially rescued.

To examine the effect of MC activation on the cell junction of BMVECs, we investigated the expression of MMP and TJ proteins, including MMP2, MMP9, ZO-1, occludin and claudin-5 using Western blot. As shown in **Figures 6C, D**, the protein expression levels of MMP2 and MMP9 in bEnd.3 cells were up-regulated and those of ZO-1, occludin and claudin-5 were down-regulated by LPS stimulation. In the P815-bEnd.3 co-culture system, these protein expression changes were seriously augmented. Immunofluorescence staining for ZO-1 and occludin showed that their fluorescence intensity was weak in the LPS-treated cells, demonstrating the broken EC junctions (**Figure 6E** with quantification in **Figure 6F**). Consistent with the *in vivo* results, cromolyn treatment for the co-culture system decreased the protein level of MMPs and increased the protein expression of TJs. All these results revealed that the activation of LPS-induced MCs can affect the cell viability and cell junctions of BMVECs, thus disrupting the normal functions of BMVECs.

### 3.7 LPS induced H1R expression and enhanced histamine responsiveness in BMVECs through the TLR2/4-MAPK signaling pathway

**Figure 7:**
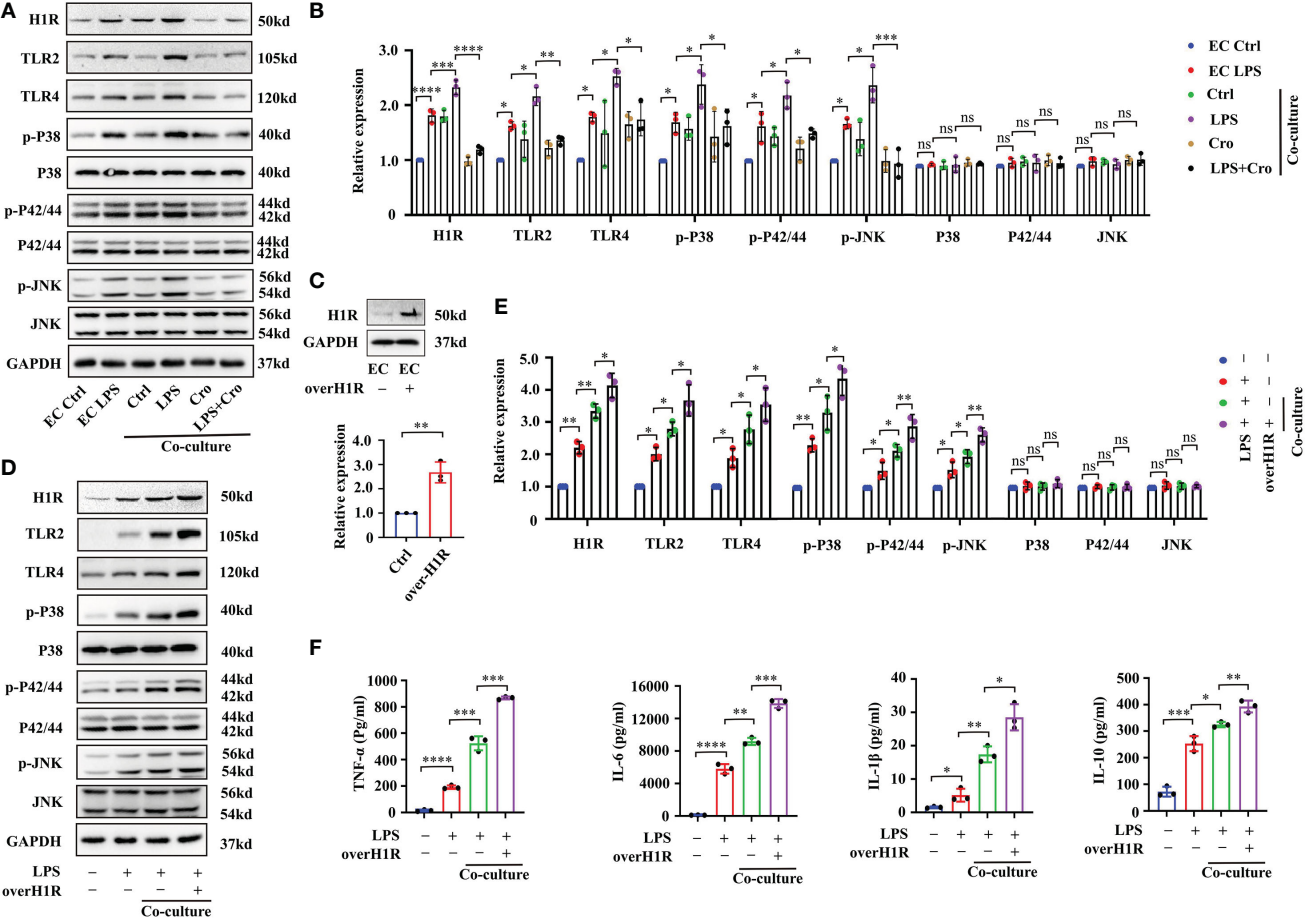
MC-secreted histamine amplified the LPS-induced inflammatory response in BMVECs through the H1R-TLR2/4-MAPK signaling pathway. **(A)** Expression levels of TLR2/4 and MAPK signaling pathway proteins (P38, P42/44 and JNK) in P815-bEnd.3 co-culture system were analyzed by Western blot (n=3). **(B)** Expression levels of TLR2/4 and MAPK signaling pathway proteins (P38, P42/44 and JNK) in the P815-bEnd.3 co-culture system were quantified and normalized to GAPDH levels (n=3). **(C)** Expression levels of H1R were checked by Western blot after bEnd.3 cells were over-expressed with H1R (n=3). **(D)** Expression levels of TLR2/4-MAPK signaling pathway proteins were checked by Western blot after bEnd.3 cells were over-expressed with H1R (n=3). **(E)** Expression levels of TLR2/4-MAPK signaling pathway proteins were quantified and normalized to GAPDH levels after bEnd.3 cells were over-expressed with H1R (n=3). **(F)** ELISA showed the changes of TNF-α, IL-6, IL-1β and IL-10 in medium supernatant after bEnd.3 cells were over-expressed with H1R (n=3). LPS, Lipopolysaccharide; MCs, Mast cells; H1R, histamine 1 receptor; TLR, Toll-like receptor; MAPK, Mitogen-activated protein kinases; (ns, no significance, *p <0.05, ** *p <*0.01, *** *p <*0.001, **** *p <*0.0001).

**Figure 8:**
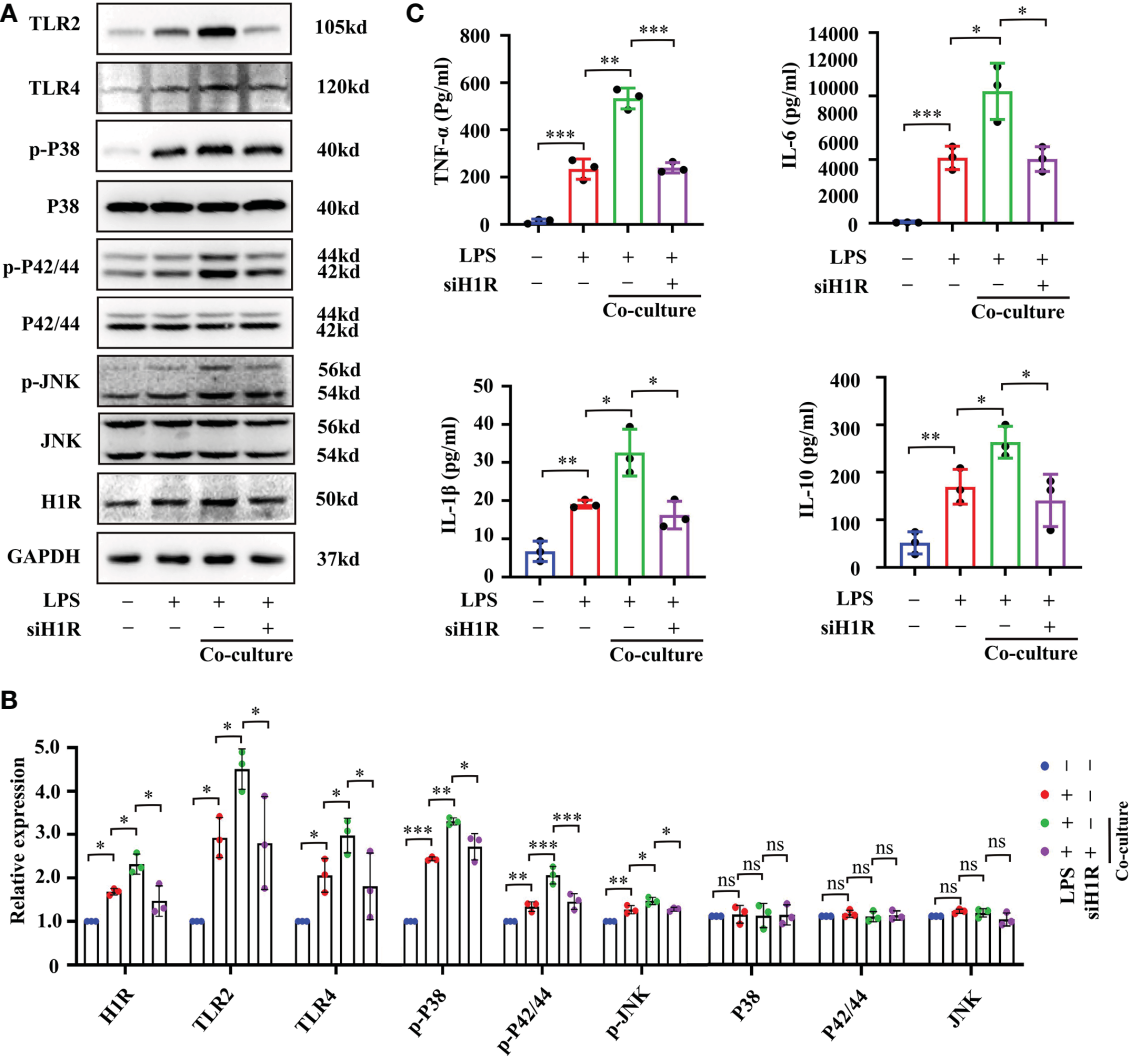
Interfering H1R expression in bEnd.3 cells decreased cell inflammation response. **(A)** Expression levels of TLR2/4-MAPK signaling pathway proteins were checked by Western blot after bEnd.3 cells were transfected with H1R siRNA (n=3). **(B)** Expression levels of TLR2/4-MAPK signaling pathway proteins were quantified and normalized to GAPDH levels after bEnd.3 cells were transfected with H1R siRNA (n=3). **(C)** Abundance of TNF-α, IL-6, IL-1β and IL-10 in medium supernatant was examined by ELISA after bEnd.3 cells were transfected with H1R siRNA (n=3). H1R: histamine 1 receptor, TLR: Toll-like receptor, MAPK: Mitogen-activated protein kinases. (ns, no significance, *p <0.05, ** p <0.01, *** p <0.001).

In human gingival fibroblasts, previous studies have confirmed that histamine promotes the expression of the receptors TLR2 and TLR4 and amplifies sensitivity to LPS treatment (27). Approximately 50% of brain histamine is produced by MCs, and H1R is highly expressed in EC. Thus, we hypothesized that MC induce BMVEC dysfunction through the histamine/H1R pathway. To test this hypothesis, we firstly checked the expression of H1R, TLR2, TLR4 and downstream mitogen-activated protein kinases (MAPK) signaling proteins by Western blot. As shown in **Figures 7A, B**, LPS treatment for 24h up-regulated H1R, TLR2 and TLR4 expression and increased the phosphorylation of P38, P42/44 and JNK in bEnd.3 cells. These protein expression changes were severely augmented in the P815-bEnd.3 co-culture system but ameliorated by cromolyn pre-treatment. To determine whether MCs promote BMVECs inflammation response mediated by histamine/H1R, we overexpressed H1R in bEnd.3 cells (**Figure 7C**). After exposure to LPS, the expression of TLR2, TLR4 and the phosphorylation of P38, P42/44 and JNK markedly increased in the bEnd.3 cells over-expressing HIR (**Figure 7D** with quantification in **Figure 7E**). To observe the inflammatory effect of P815-bEnd.3 cells co-cultures, we detected IL-6, IL-1β, TNF-α and IL-10 in the supernatant using ELISA and found that the abundance of cytokines was gravely increased in the bEnd.3 cells over-expressing HIR (**Figure 7F**). We used the specific siRNA to silence the expression of H1R in bEnd.3 cells. Adverse effects were found as shown in **Figures 8A–C**. These data supported that histamine/H1R mediates the interaction between MCs and BMVECs and amplifies the LPS-induced inflammatory response in BMVECs *via* the TLR2/4-MAPK signaling pathway.

## 4 Discussion

In this study, we used a classical animal model of sepsis, CLP, to induce SAE. Consistent with previous findings, CLP successfully induced SAE and systemic inflammation (28). We further confirmed that MCs were excessively activated under SAE, and their activation triggered a severe inflammation cascade that weakened BBB integrity by disrupting TJ proteins, leading to impaired learning and memory function. These sepsis-induced changes were alleviated by cromolyn (a most characteristic MCs stabilizer), further supporting the important role of MCs in SAE and suggesting the therapeutic effect of cromolyn on SAE. In an *in vitro* model of LPS-induced inflammation, we found that MC-secreted histamine-mediated BMVEC activation and amplified the endothelial inflammatory response *via* the TLR2/4-MAPK signaling pathway.

Sepsis is a life-threatening organ dysfunction caused by immune dysregulation in response to an infection. SAE represents diffuse cerebral dysfunction during sepsis, and the incidence of SAE in patients with sepsis is about 9%~70%, thus seriously affecting the prognosis of patients with sepsis (3);. Clinically, patients with SAE exhibit acutely altered mental status and usually have higher mortality and morbidity than those without SAE (29). Several SAE animal models have been established, such as CLP and LPS intraperitoneal (i.p) injection (30, 31). Although LPS-induced endotoxemia is frequently used to mimic sepsis, the CLP is considered as a clinically relevant model with polymicrobial peritonitis induced by the cecal puncture and necrotic tissues induced by cecal ligation. However, the CLP model can produce largely variable results, with survival rates ranging from 20% to 50% in 24 h following CLP in rats. Singleton et al. demonstrated that the length of the cecum ligated is a major determinant of mortality in the CLP model of sepsis (24). Their findings indicated that the mortality and inflammation data from this model can be adjusted to fit the individual needs of a particular experiment. Therefore, in our pre-experiment, we generated 25%, 50% and 75% of the cecal length of mice that were ligated and examined the mortality, body weight change and cytokines content. Our results found that 75% of the cecal lengths ligated group induced a mortality rate of over 50% within 7 days and a significant decrease in body weight and obvious release of cytokines, which is consisted with previous studies (22, 32, 33). Considering that SAE is associated with severe neuroinflammation responses, cognitive dysfunction and significant mortality, we finally determined 75% of the cecal length ligated used for further investigation. In the next experiments, ELISA results showed that the production of the inflammatory cytokines (TNF-α, IL-6, IL-1β and IL-10) in the hippocampus homogenate markedly increased in the CLP mice than those of sham mice. In addition to the severe inflammatory response, the surviving septic mice also exhibited severe exploration learning and memory impairments as demonstrated by open field trials and the Y maze. This finding implied that the CLP mice are a reliable SAE model for further study.

SAE has multiple mechanisms, including systemic inflammation, cerebrovascular dysfunction, BBB damage, neuroinflammation, oxidative stress, and excessive microglia and astrocyte activation (34, 35). Among them, neuroinflammation and BBB dysfunction are the most important components in the pathogenesis of SAE. Microglial cells are the dominating effector and the early inflammatory responder in neuroinflammation. During sepsis, endotoxemia and pro-inflammatory cytokines are released systemically, leading to excessive microglial activation and mass production of inflammatory cytokines and perpetuating a vicious cycle that causes brain impairment and contributes to the progression of SAE (31). Accumulating evidence has indicated that MCs activation plays a vital role in allergic reactions, and a variety of CNS diseases, such as stroke, brain trauma and neurodegenerative diseases. Existing research suggests that activated MCs could migrate rapidly to the site of injury and degranulate a few seconds after crosslinking, resulting in the release of prestored inflammatory mediators (36) which facilitate the recruitment of immune cells (neutrophils, lymphocytes, eosinophils, basophils) and the amplification of inflammatory response (37). In the rat HI model, researchers found that MCs are the first cells to respond to HI in the immature brains (38). Thus, inhibition of this early MCs response is sufficient to provide long-term protection. Although other resident cells in the CNS produce TNF-α, most notably microglia/macrophages and ECs, the presence and release of this important cytokine from the MCs preceded its detection in other cells (38). In the model of LPS-induced neuroinflammation, MC was the earliest participant, activated 2h after LPS stimulation, while microglia became significantly activated at 4 h (10). Thus, researchers defined MCs as the first responders of injury in the CNS (10). Additionally, MCs could release newly formed mediators in the next several days and further amplify inflammatory responses in the CNS (39). Therefore, we firstly hypothesized that MCs activation involves in sepsis-induced neuroinflammation. To test our hypothesis, we used TB and tryptase antibody for MC staining. The number and activation status of MCs were significantly increased in the hippocampus section of SAE mice. The degranulated MCs showed irregular shapes with extensive cytoplasmic granules in the extracellular layer. To further confirm the role of MCs in SAE, we used cromolyn to treat the CLP-induced SAE mice. Cromolyn is a well-studied stabilizer of MCs. Although a research published by Oka et al. (40) suggested that there are potential off-target/mast cell-independent anti-inflammatory effects of cromolyn in allergic mice. Numerous previous studies confirmed that cromolyn can inhibit MCs-associated inflammation responses, and suggested cromolyn as a valuable therapeutic agent in MCs-involved neurological diseases (6–9). Consistent with previous studies, our results showed that cromolyn sharply reduced the number of activated MCs, inhibited the release of inflammatory cytokines in the hippocampus, decreased the mortality rate and alleviated the cognitive impairment of septic mice. This finding suggested that the neuroinflammation of septic mice is partly associated with MCs activation.

The BBB is a highly selective and semipermeable interface between the cerebral parenchyma and the blood that is composed of BMVECs, pericytes, astrocytes, microglial cells and TJs between the BMVECs. Under a physiological context, the BBB helps maintain brain homeostasis by restricting the movement of harmful substances and cells between the blood and brain (41, 17, 31). Increasing evidence indicates that the integrity and permeability of BBB are impaired during sepsis (5, 41). Loss of BBB integrity is a key cause of sepsis-induced cerebral dysfunction, that is, many immune cells and neurotoxic mediators may directly enter the CNS. Our results confirmed that BBB permeability is increased in septic mice as demonstrated by BWC, FS and EB extravasation. BMVECs and TJs between them are the most important structures that maintain BBB integrity (42). The dysfunction of BMVECs and the reduction in TJ proteins can affect BBB integrity and lead to deficits in BBB function. In human brain tissue samples from deceased patients with sepsis, a remarkable downregulation of TJ proteins (occludin, claudin-5 and ZO-1) was found in BMVECs, demonstrating impaired BBB integrity (43). MMPs (especially MMP-9 and MMP-2), which are significant components of ECM proteasomes, play an important role in BBB leakage by degrading TJ proteins ZO-1 and occludin (25). In this work, we firstly checked the expression of TJ proteins ZO-1 and occludin and the expression of MMP2 and MMP9. The results showed a significant decrease in ZO-1 and occludin and an up-regulation of MMP2/9 in the hippocampus of SAE mice. Similar results were also found in the LPS-induced cell inflammation model. Cromolyn significantly alleviated the degradation of TJ proteins and inhibited the increase in MMP2/9, thus rescuing BBB disruption. In summary, activated MCs mediate BBB dysfunction in SAE mice, and the inhibition of MC activation protects BBB permeability and integrity.

Although BMVECs are the basic component of BBB, direct interactions between MCs and BMVECs are poorly studied. During sepsis, activated BMVECs express various adhesion molecules, including CD40, e-selectin, VCAM, ICAM, and inflammatory receptors, such as IL-1, TNF-α and TLR4, which facilitate the infiltration of leukocytes and inflammatory mediators into the brain parenchyma (44, 45). Additionally, the activation of BMVECs generates an intravascular inflammatory microenvironment, which induces the activation of other immune cells and the promotion of neuroinflammatory response (46). Whether MCs can trigger the activation of BMVECs and the underlying molecular mechanisms are still unknown. Through TLR4 activation, LPS up-regulates the expression and function of H1R and amplifies histamine-induced inflammatory responses in HCAECs (47). In HUVECs, histamine up-regulates the expression of TLR2 and TLR4 and amplifies EC inflammatory responses to Gram-negative and Gram-positive bacterial components (20). Similarly, a previous study also confirmed that histamine also promotes the expression of receptors TLR2 and TLR4 and amplifies sensitivity to LPS treatment in human gingival fibroblasts (27). Histamine is a neurotransmitter and an immune modulator, and both of these roles are involved in multiple biological processes. In a classic allergic reaction, histamine activates H1R on ECs, increasing vascular permeability and activating smooth muscle cells that lead to contraction (48). As an inflammatory mediator, histamine exposure can induce microglia into a pro-inflammatory state, which has deleterious effects on the brain (10, 49). MCs are an important source of histamine in the brain, accounting for up to 50% of brain histamine levels in rodents (6, 50). Thus, the modulation of inflammation by histamine in CNS may be linked to MCs activation. We hypothesized that the sepsis-induced activation of MCs may facilitate the inflammatory responses of BMVECs *via* the histamine pathway. Thus, we firstly checked the expression of H1R, TLR2, TLR4 and typic downstream MAPK signaling pathways (P38, P42/44 and JNK). The results showed that LPS-induced MCs activation could stimulate the expression of H1R, TLR2 and TLR4, which then activated the MAPK signaling pathway, promoted the release of inflammation mediators from BMVECs and ultimately amplified the inflammatory response. With further interference and overexpression experiments, the findings suggested that the MC-released histamine mediates the amplification of the inflammatory response in BMVECs by binding to H1R.

To the best of our knowledge, this study is the first demonstration of sepsis-induced MCs activation in SAE. We found that early treatment with the MCs stabilizer, cromolyn can provide a neuroprotective effect by weakening the neuroinflammation cascade. This work provides preliminary evidence for the protective role of cromolyn in SAE. SAE is a significant clinical issue. If the protective effect of cromolyn on SAE can be confirmed in clinical trials, then the translation of our findings into clinical practice could be profound.

## Data availability statement

The original contributions presented in the study are included in the article/**Supplementary Material**. Further inquiries can be directed to the corresponding authors.

## Ethics statement

The animal study was reviewed and approved by The Institutional Animal Care and Use Committee of Guizhou Province People’s Hospital (Guizhou, China).

## Author contributions

YT and JZ designed the experiments, edited the manuscript and provided research funding. JY performed the experiments, acquired the data, analyzed the data and drafted the manuscript. RH and MD acquired and analyzed the data. JG and SY collected, analyzed and verified the data. YX, GH, LL, JL, YC and YZ conceived the idea and edited the manuscript. All authors contributed to the article and approved the submitted version.

## Supplementary material

Supplementary Figure 1 The survival rate, body weight, and hippocampal inflammatory factors production in mice with 25%, 50%, and 75% of the cecal lengths ligated. **(A)** Survival rate was observed in 7 days following surgery (n=15). **(B)** Body weight was observed in 7 days following surgery (n=6); **(C–F)** The levels of TNF-α, IL-6, IL-1β and IL-10 in mice hippocampus tissues were detected by ELISA (n=6). (ns: no significance, **p <*0.05, ** *p <*0.01, *** *p <*0.001, **** *p <*0.0001).

Supplementary Figure 2 Sepsis-induced systemic inflammation and cognitive deficits. **(A)** Levels of TNF-α, IL-6, IL-1β and IL-10 in mice serum were detected by ELISA (n=6). **(B, C)** Movement trace of mice in the Y maze test (n=6). (** *p <*0.01, *** *p <*0.001, **** *p <*0.0001).
