# Supplementary figures and images for: Mast cell activation mediates blood–brain barrier impairment and cognitive dysfunction in septic mice in a histamine-dependent pathway
Source: Front Immunol. 2023 Feb 1;14:1090288. doi: 10.3389/fimmu.2023.1090288 (PMC9929573; doi:10.3389/fimmu.2023.1090288)

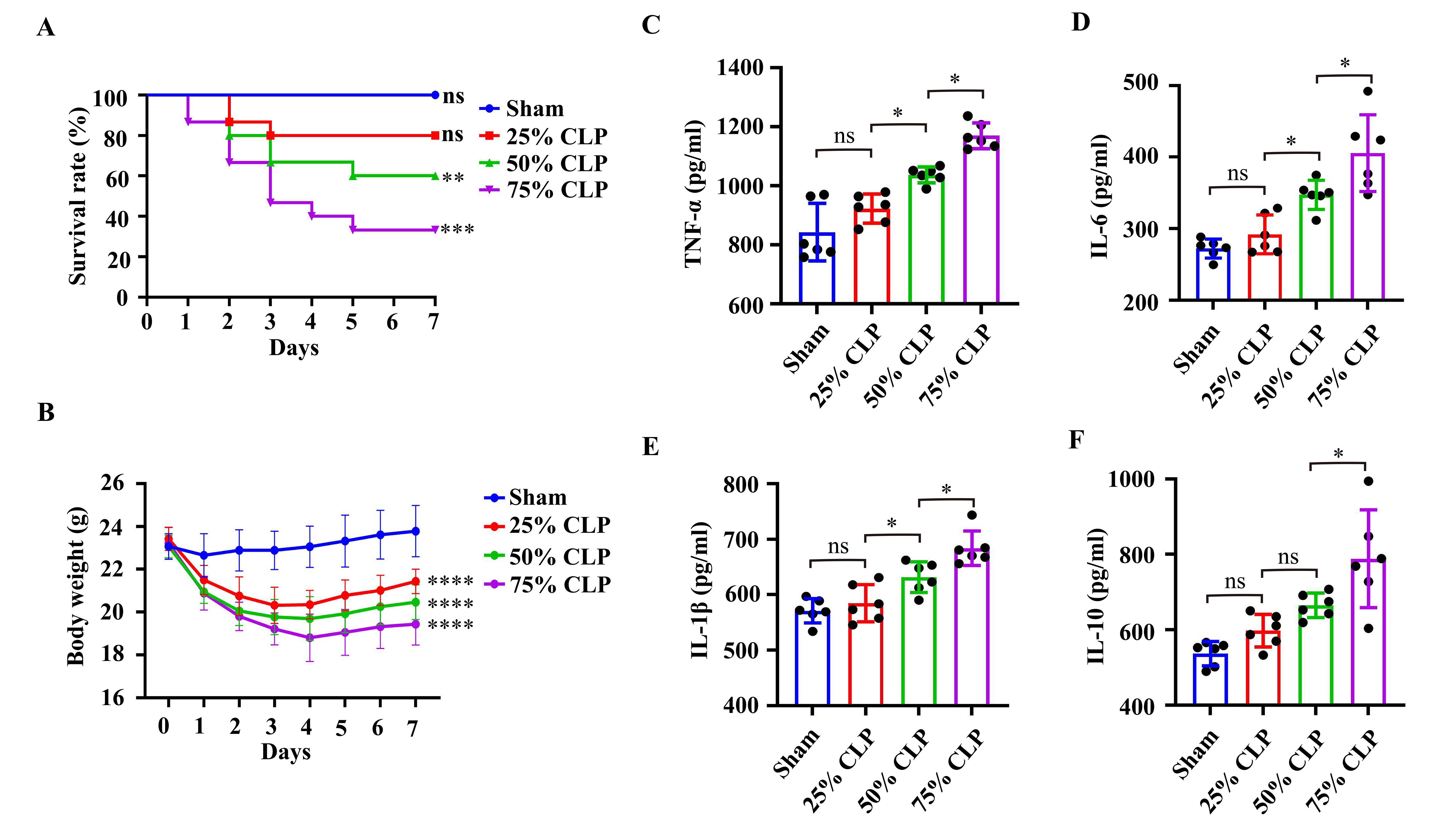

Supplement: Supplementary Figure 1 — The survival rate, body weight, and hippocampal inflammatory factors production in mice with 25%, 50%, and 75% of the cecal lengths ligated. (A) Survival rate was observed in 7 days following surgery (n=15). (B) Body weight was observed in 7 days following surgery (n=6); (C–F) The levels of TNF-α, IL-6, IL-1β and IL-10 in mice hippocampus tissues were detected by ELISA (n=6). (ns: no significance, *p <0.05, ** p <0.01, *** p <0.001, **** p <0.0001). [file Image_1.tif]

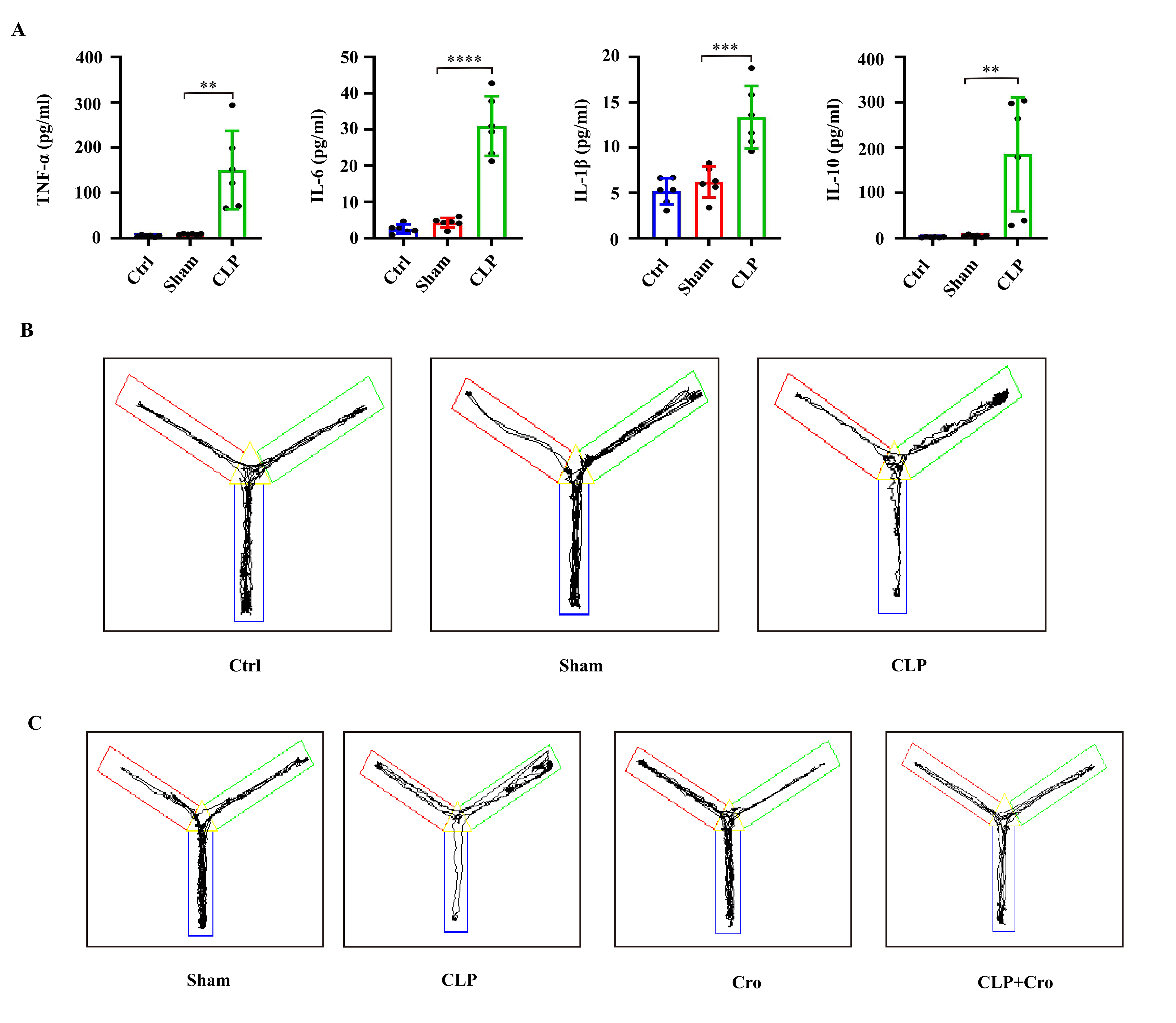

Supplement: Supplementary Figure 2 — Sepsis-induced systemic inflammation and cognitive deficits. (A) Levels of TNF-α, IL-6, IL-1β and IL-10 in mice serum were detected by ELISA (n=6). (B, C) Movement trace of mice in the Y maze test (n=6). (** p <0.01, *** p <0.001, **** p <0.0001). [file Image_2.tif]
