# Supplementary material for: Mast cell activation mediates blood–brain barrier impairment and cognitive dysfunction in septic mice in a histamine-dependent pathway
Source: Front Immunol. 2023 Feb 1;14:1090288. doi: 10.3389/fimmu.2023.1090288 (PMC9929573; doi:10.3389/fimmu.2023.1090288)
Supplement: Supplementary file 3 [file Table_1.docx]

| **Supplementary table 1. List of antibodies or reagents.** | | | | |
| --- | --- | --- | --- | --- |
| **Reagents or antibodies** | **Vendors** | **Country of vendors** | **Catalog numbers** | **Concentrations or dilution ratio** |
| Cromolyn | Sigma-Aldrich | Germany | C0399 | 10mg/mL |
| Compound 48/80 | Sigma-Aldrich | Germany | C2313 | 10mg/mL |
| Lipopolysaccharide | Sigma-Aldrich | Germany | L2630 | 1mg/mL |
| DMEM | Gibco | USA | 11995040 | - |
| Fetal bovine serum | Gibco | USA | 10-099141 | 10% |
| Penicillin/streptomycin | Beyotime | China | C0222 | 1% |
| Polybrene | Solarbio | China | H8761 | 8 µg/mL |
| Puromycin | Beyotime | China | ST551 | 1 ug/mL |
| RNAi max | Invitrogen | USA | 13778030 | - |
| TNF-α ELISA kit | Boster | China | EK0527 | - |
| IL-6 ELISA kit | Boster | China | EK0411 | - |
| IL-1βELISA kit | Boster | China | MEK101 | - |
| IL-10 ELISA kit | Boster | China | EK0417 | - |
| Histamine ELISA kit | Elabsecience | China | E-EL-0032c, | - |
| Tryptase ELISA kit | Meimian | China | 14547 | - |
| Lysis buffer | Solarbio | China | R0010 | - |
| ECL kit | Milipore | USA | WBKLS0100 | - |
| Fluorescein sodium | Sigma-Aldrich | Germany | F6277 | 2% |
| Evans blue | Sigma-Aldrich | Germany | E2129 | 2% |
| Toluidine Blue | Solarbio | China | G3661 | 1% |
| Bovine serum albumin | Beyotime | China | ST2249 | 10% or 5% |
| Triton X-100 | Solarbio | China | T8200 | 0.3% |
| DAB | Solarbio | China | DA1016 | - |
| Hematoxylin | Solarbio | China | H8070 | - |
| ZO-1, | Abcam | UK | ab221547 | 1:100 or 1:1000 |
| Occludin, | Abcam | UK | ab216327 | 1:100 or 1:1000 |
| Ki67 | Abcam | UK | ab16667 | 1: 100 |
| Tryptase | Abcam | UK | Ab2378 | 1: 100 |
| Phospho-MAPK Family | CST | USA | 9910T | 1: 1000 |
| MAPK Family | CST | USA | 9926T | 1: 1000 |
| TLR2 | Abcam | UK | ab209217 | 1: 1000 |
| TLR4 | Absin | China | abs13200 | 1: 1000 |
| MMP9 | Abcam | UK | ab283575 | 1: 1000 |
| MMP2 | CST | USA | 87809S | 1: 1000 |
| GAPDH | CST | USA | 5174S | 1: 3000 |
| H1R | Santa | USA | sc-374621 | 1: 500 |
| Claudin-5 | Santa | USA | sc-374221 | 1: 500 |
| Anti-rabbit IgG | CST | USA | 14708 | 1: 2000 |
| Anti-mouse IgG | CST | USA | 14709 | 1: 2000 |
| Goat anti-mouse Alexa Fluor Plus 488 secondary antibody | Invitrogen | USA | A32732 | 1: 1000 |
| DAPI | Invitrogen, | USA | S36920 | - |
